# Editorial

**Published:** 1989-08

**Authors:** 


### Editorial

**Richard Bright**

It may come as a surprise to many Bristolians to learn that Richard Bright was a son of Bristol. He was born 200 years ago into a family prominant for generations in Bristol affairs and spent his childhood in the idyllic surroundings of Ham Green House. To celebrate the occassion Dr Campbell Mackenzie, Director of the Renal Unit at Southmead Hospital and Secretary of the Richard Bright Society has written a biographical article about this 'man of many parts'. His Bristol connection was no more than an accident of birth, he was a graduate of Edinburgh University and it was at Guy's that his life's work was done, nevertheless we can still take pleasure if not pride in his Bristol association and we dedicate this number to his name.

**Alternative Treatment for Sex Offenders**

Though justice may be done in the incarceration of sex offenders the chief value to society lies in keeping them out of harm's way for a period of time. When sooner or later they are released the likelihood that they will reoffend is high. In an important article in this number Mr Clive Gingell and Mr Hugh Gilbert advance the argument that new drugs used in the treatment of carcinoma of the prostate which remove the 'sex drive' could also be used to render such individuals harmless on release. If society can accept this form of chemical castration our woman will be safer our prisons less overcrowded.

**West of England Medical Journal on course**

This matter has been discussed at length in previous editorials. A meeting was held recently at Moretonhampstead. Delegates from a number of medical groups in the West of England met to discuss the desirability of forming an association to produce a broadly based successor to this journal. Agreement was reached on the aims and the methods to be pursued. The delegates will report back to their societies and if the project receives sufficient backing, with the sponsorship of the Nuffield Nursing Homes Trust we can start in 1990.

